# Podoplanin is a useful prognostic marker and indicates better differentiation in lung squamous cell cancer patients? A systematic review and meta-analysis

**DOI:** 10.1186/s12885-020-06936-9

**Published:** 2020-05-14

**Authors:** Liya Hu, Peng Zhang, Qi Mei, Wei Sun, Lei Zhou, Tiejun Yin

**Affiliations:** 1 Department of Geriatrics, Tongji Hospital, Tongji Medical College, Huazhong University of Science and Technology, Jiefang Avenue 1095, Wuhan, 430030 Hubei China; 2 Department of Oncology, Tongji Hospital, Tongji Medical College, Huazhong University of Science and Technology, Jiefang Avenue 1095, Wuhan, 430000 Hubei China

**Keywords:** Lung squamous cell carcinoma, Meta-analysis, PDPN protein, Stem cell marker, Prognosis

## Abstract

**Background:**

The CSC (cancer stem cell) markers often indicate poor prognosis and more cell invasion or migration of cancer patients. Podoplanin was assumed as a candidate CSC marker and predict poor prognosis among squamous cancers. Whereas, the prognostic value of podoplanin among lung squamous cancer (LUSC) patients remains controversial.

**Methods:**

A search of databases including PubMed, Embase and Web of Science was performed. Eligible articles studying the prognostic significance of podoplanin were selected. Odds ratio and HR (hazard ratio) were used to assess the relationships between podoplanin and clinical characteristics, as well as to quantify its prognostic role. The heterogeneity was estimated by I^2^ Statistic and *P* values from sensitivity analysis. Begg’s funnel plots were used to estimate possible publication bias.

**Results:**

8 eligible studies containing 725 I-IV LUSC patients were included. Podoplanin expression showed no significant correlations with TNM stage, vascular invasion, lymphatic invasion, lymph node metastasis, pleural metastasis of tumor and gender of patients. However, podoplanin showed significant associations with better differentiation (pooled OR = 2.64, 95% CI 1.53–4.56, *P* = 0.0005, fixed effect) and better overall survival (HR = 2.14, 95% CI 1.45–3.15, *P* = 0.0001, fixed effect) and progression-free survival (HR = 1.73, 95% CI: 1.01–2.98, *P* = 0.05, fixed effect) of LUSC. Funnel plots illustrated no evidence of publication bias in our results.

**Conclusions:**

Podoplanin could be a useful prognostic marker and indicates better differentiation for LUSC patients, and the value of PDPN expression as a marker for cancer stem cells in LUSC should be critically evaluated in future studies.

## Background

Lung cancer is the leading cause of cancer mortality across the world. Progress in molecular markers have been increasingly reported to predict prognosis and survival of patients with non-small cell lung cancer (NSCLC) [1]. However, lung squamous cell carcinoma (LUSC), as one of the main type of lung cancer, has not much progress in the molecular targeted treatment compared with adenocarcinoma, and the 5-year survival rate is still less than 20% [2].

Cancer stem cells (CSCs) are a small subpopulation of cells within tumors with capabilities of self-renewal, differentiation, and tumorigenicity, which usually associated with resistance to therapy and poor prognosis in clinical outcomes [3]. Reports have identified certain gene signatures and biomarkers to characterize CSCs in different tumor types. Podoplanin (PDPN) is a 38 kDa mucin-like type I transmembrane protein which expressed in multiple tissues during ontogeny, including the brain, heart, kidney, lungs, osteoblasts, and lymphoid organs [4, 5]. Recently, it is reported that it also appears in tumors, especially in squamous cell cancers (SCC), such as lung cancer [6, 7], malignant mesothelioma [8], head and neck squamous cell cancers [9], uterine cervix carcinoma [10] and so on. Several studies also showed evidences of PDPN in regulating stem cells in normal and tumor tissues. In normal tissues, PDPN involves in the control of the mammary stem-cell function by impaired its growth and self-renewal potential due to downregulation of Wnt/β-catenin signaling activity [11]. In glioma, PDPN is considered as a novel marker of glioma-dervied cancer stem cells for the low sphere formation rates and resistance to ionizing radiation in the PDPN-positive group [12] . In vivo and vitro experiments among SCC, several evidences showed that PDPN-positive cells have higher colony formation and tumorigenicity, which may act as a candidate CSC marker [13, 14].

While PDPN showed disparate correlations with lymph nobe metastasis and survival rates among different kinds of squamous cancer patients [15, 16]. For instance, in cutaneous squamous cell carcinoma (cSCC), PDPN is significantly upregulated in metastatic (*p* = 0.002) and poorly differentiated (*p* = 0.003) cancer patients [17]. However, Kimberly L Dumoff showed that PDPN expression in pretreatment biopsy material predicted better prognosis in advanced-stage squamous cell carcinoma of the uterine cervix [10]. Thus, PDPN seems to have two faces as a potential therapeutic target among different squamous tumors [18].

In lung squamous cell cancer, recent studies have produced controversial results regarding the clinical prognostic role of PDPN in LUSC. Liyi Xie demonstrated high PDPN expression significantly associated with worse clinicopathological features (pleural invasion, et al) and worse progression-free survival (PFS) [19]. Kyuichi Kadota demonstrated that PDPN is a significant prognostic factor of poor prognosis for LUSC patients [20]. Whereas, other studies like Yoshihisa Shimada reported that patients with PDPN^+^ lung squamous cancers resulted in significant better overall survival (OS) [21]. Hence, the prognostic role of PDPN in LUSC is still obscure. In order to clarify the associations between PDPN and clinicopathological features and its prognosis value among squamous lung cancer patients, we performed a systematic review and meta-analysis of the published researches.

## Methods

### Literature search strategy

We conducted a comprehensive systematic literature search of online database including PubMed, Embase and Web of Science from 2000 to 2019 identify all observational or retrospective studies. Search terms and relative variants included: podoplanin, PDPN, D2–40, aggrus, T1alpha, GP36, OTS8, survival outcome, overall survival, prognosis, lung squamous cell cancer, SqCC, LUSC. We also reviewed the references of included articles and related systematic reviews to identify additional related studies. This review has been submitted at PROSPERO on 10th of Dec, 2019 (ID:161923), and it is now under assessment.

### Selection criteria

The inclusion criteria were as follows: (I) studies had to conducted on squamous cell lung cancer patients; (II) the correlations between the expression and prognosis of PDPN has been reported; (III) PDPN expression level was measured by immunohistochemistry (IHC); (IV) the hazard ratios (HRs) and 95% confidence intervals (CIs) could be extracted directly or calculated indirectly; (V) published in English.

The Newcastle-Ottawa Scale (NOS) star system (range, 0–9 stars) was used to assess the quality of the included studies and was performed by two team members (Peng Zhang and Wei Sun) independently. Differences were discussed to achieve consensus by a third team member (Qi Mei). For no standard criteria has been established, 6 or more stars were considered as a high-quality study in our current study.

### Data extraction

Data extraction was independently conducted by two independent investigators (Zhang and Zhou). Any disagreement was resolved by another investigator (Qi Mei). A data extraction sheet based on the Cochrane Consumers and Communication Review Group’s data extraction template was utilized. The following details were extracted: (I) details of the study: first author, publication year, country of patients and sample size; (II) clinicopathological features: race, gender, tumour TNM stage, vascular invasion, lymphatic invasion, lymph node invasion, pleural metastasis, location of protein expression; (III) Survival analysis related features: the proportion and patient number of positive PDPN expression, cut-off standard for the definition of positive staining or staining intensity, follow-up time and survival data (OS and PFS). Two reviewers (Zhang and Zhou) collected the data independently from every eligible study. Any unclarity or lack of disagreement was resolve by discussion with a third reviewer until final consensus.

### Statistical analysis

For each applicable study, the HR and the corresponding 95% confidence intervals (CIs) were used to evaluate the association between PDPN expression and survival outcomes of OS and PFS. Data of HR and 95% CI were extracted from the original studies or from available survival curves by the Tierney’s methods if the data (HR and 95% CI) were not reported [22]. ORs and 95% CI were used to evaluate the correlations among PDPN expression and the clinicopathological features for squamous cell lung cancer patients, which included the vascular invasion, lymphatic invasion, lymph node metastasis, pleural metastasis, differentiation of tumor and gender of patients. The heterogeneity across the studies was estimated by I^2^ Statistic and *P* values. ORs and HRs were evaluated with random-effect model when the I^2^ was more than 30% and P value was less than 0.05. Otherwise, a fixed-effect model was conducted. The influence of the heterogeneity of individual studies was displayed when deleting each study at one time by sensitivity analysis. Furthermore, Begg’s funnel plots were used to estimate possible publication bias [23]. A value of *P* value less than 0.05 was considered to be potential publication bias. Cochrane Review Manager version 5.3 (Cochrane Library, Oxford, UK) was used to calculate the ORs and HRs and their variations from each investigation.

## Results

### Quality assessment and description of the included studies

**Fig. 1:**
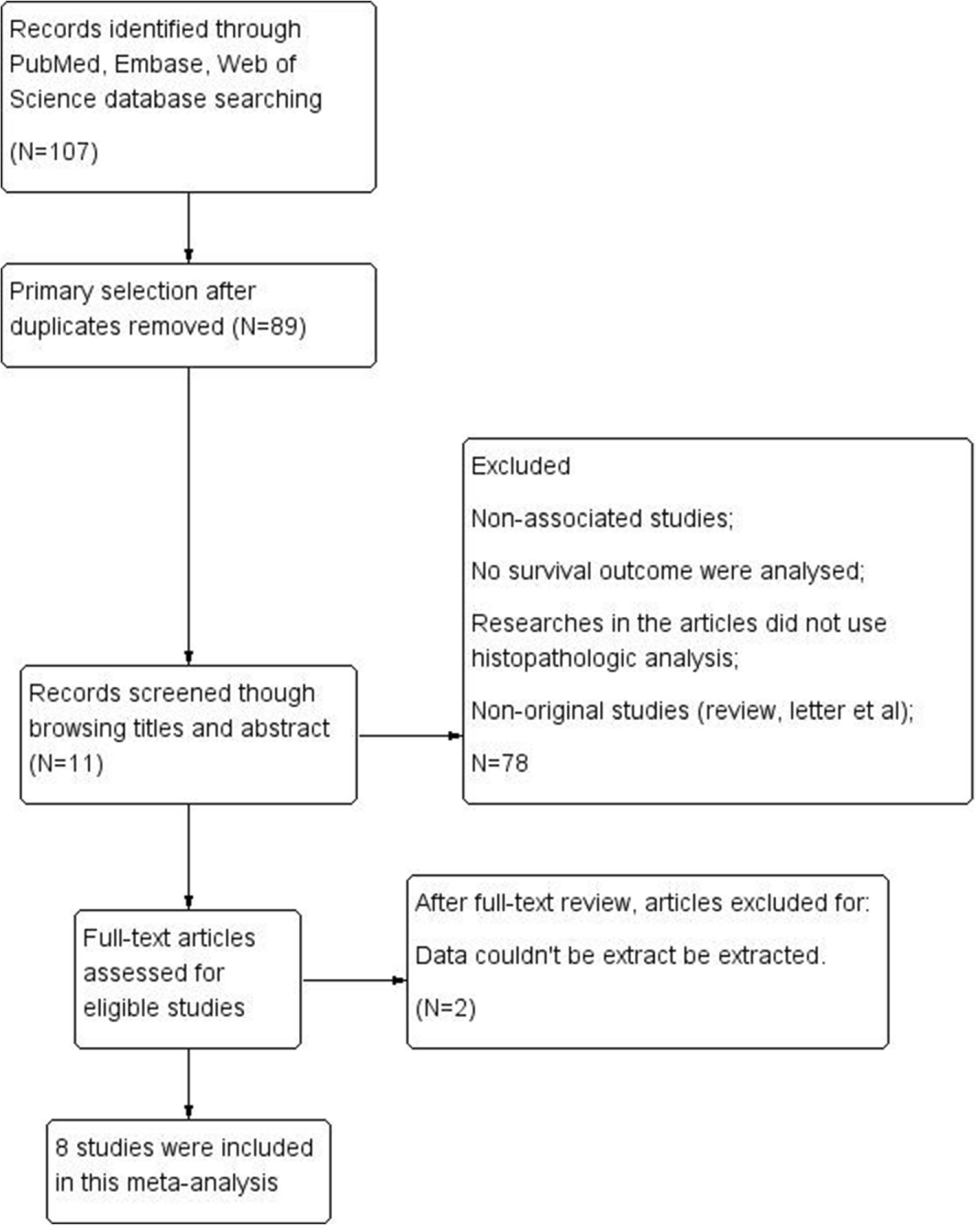
Literature search strategy and selection of articles

A total of 107 articles were retrieved through the database search from PubMed, Embase and Web of Science, of which 89 references remained after duplicate screening. After title and abstract assessment, 78 references were excluded according to the inclusion criteria. 11 references were found eligible. Finally, through full-text evaluation, 8 studies contained the data of OS or PFS, which were suitable for this meta-analysis (Fig. 1). The reasons for excluded studies were: (1) studies were not associated with survival of clinical research; (2) PDPN expression was not assessed by immunohistochemistry; (3) PDPN was expressed on non-tumor cells; (4) survival data couldn’t be extracted either from the articles nor by Tierney’s methods described above; (5) non-original articles. The quality of individual studies were evaluated through NOS quality assessment tool. The maximum score was 9 stars: 4 for selection, 2 for comparability and 3 for outcomes. Finally, the mean value for the 8 studies was 6 stars (Table 1). Among them, 7 studies contain OS data, and 3 studies contain PFS data. In summary, a total number of 725 I-IV LUSC patients were included in our current study. All the 7 articles dealt with clinicopathological factors. The characteristics and demographics of the 8 included studies are summarized in Table 1.

**Table 1:** characteristics and quality assessment of the included studies

Study	Year	Tumor type	Survival outcome	Country	Pathological stage	Number of patients		Follow-up period (mean/range, months)	Cut-off for PDPN positive	NOS Quality Score
						PDPN+	PDPN-			
Hanako Suzuki	2011	LUSC	OS,PFS	Japan	I–III	16	24	40	>25%	7
Juan Li	2017	LUSC	OS	China	I-IV	60	22	19.5	>80%	7
Kyuichi Kadota	2010	LUSC	OS	Japan	I–III	12	38	till 80	>30%	8
Liyi Xie	2018	LUSC	PFS	China	I-IV	28	42	53.4	>75%	6
Takeo Ito	2008	LUSC	OS,PFS	Japan	IB	69	67	43.9	>10%	8
Shotaro Iwakiri	2009	LUSC	OS	Japan	I–IIIA	40	42	60	weak and strong, none staining	6
Yoichiro IKOMA	2015	LUSC	OS	Japan	I–III	32	71	51.7	≥10%	8
Yoshihisa Shimada	2009	LUSC	OS	Japan	I–III	107	55	60	>10%	8

*ND* not document, *IHC* immunohistochemistry

### Correlation of PDPN expression with Clinicopathological parameters

**Fig. 2:**
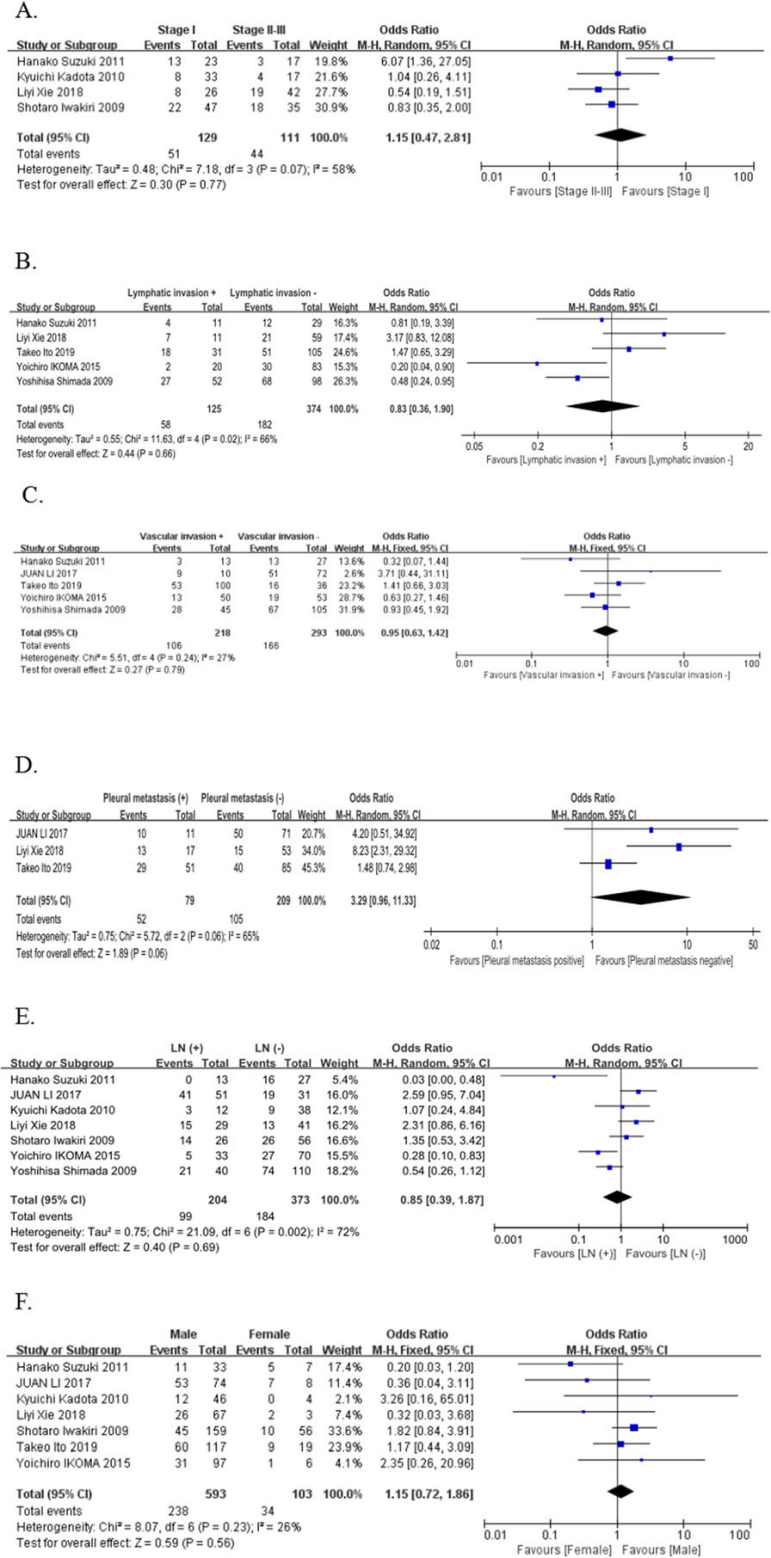
Forest plot depiction of podoplanin expression and OR for clinical pathologic features. Clinicopathological parameters investigated are TMN classification (**a**), lymphatic invasion (**b**), vascular invasion (**c**), pleural metastasis (**d**), lymph node metastasis (**e**), sex (**f**). OR with corresponding confidence intervals are shown

The distribution of different parameters (vascular invasion, lymphatic invasion, lymph node status, pleural metastasis and Stage) in PDPN positive and negative groups were summarized in Table 2. The association between PDPN and clinicopathological parameters is displayed in Fig. 2. PDPN expression has significantly high correlations with better differentiation of squamous cell lung carcinoma (pooled OR = 2.64, 95% CI 1.53–4.56, *P* = 0.0005, fixed effect). However, PDPN has no correlations with TNM stage (pooled OR = 1.58, 95% CI 0.53–4.69, *P* = 0.41, random effect) (Fig. 2a), lymphatic invasion (pooled OR = -0.04, 95% CI -0.23-0.14, *P* = 0.64, random effect) (Fig. 2b), vascular invasion (pooled OR = 0.95, 95% CI 0.63–1.42, *P* = 0.79, fixed effect) (Fig. 2c), pleural metastasis (pooled OR = 3.29, 95% CI 0.96–11.33, *P* = 0.06, random effect) (Fig. 2d), lymph node metastasis (pooled OR = -0.08, 95% CI -0.29-0.14, *P* = 0.49, random effect) (Fig. 2e), sex (pooled OR = 1.15, 95% CI 0.72–1.86, *P* = 0.56, fixed effect) (Fig. 2f).

**Table 2:** Demographics of the included studies

Study	Sex (female%)	Vascular invasion				Lymphatic invasion				Lymph node status								Pleural metastasis				Stage							
		PDPN positive		PDPN negative		PDPN positive		PDPN negative		PDPN positive				PDPN negative				PDPN positive		PDPN negative		PDPN positive				PDPN negative			
		+	–	+	–	+	–	+	–	N0	N1	N2	N3	N0	N1	N2	N3	+	–	+	–	I	II	III	IV	I	II	III	IV
Hanako Suzuki	20%	3	13	10	14	4	112	7	17	16	0			11	13			ND				13	3		None	10	14		None
JUAN LI	9.60%	9	51	1	21	ND				19	41			12	10			10	50	1	21	35		25		18		4	
Liyi Xie	4.30%	ND				7	14	4	38	13	7	8	0	28	11	3	0	13	15	4	38	8	9	10	1	18	16	7	1
Takeo Ito	20%	53	16	47	20	18	51	13	54	69	0			67	0			29	40	22	45	ND							
Yoichiro IKOMA	5.80%	13	19	37	34	2	30	18	53	27	5			43	28			ND				ND							
Yoshihisa Shimada	9.30%	28	67	17	38	27	68	25	30	74	21			36	19			ND		17	38	ND							
Kyuichi Kadota	8%	ND				ND				9	3			29	9			ND				8	1	3	None	25	5	8	None
Shotaro Iwakiri	9.80%	ND				ND				25	6	8	–	30	4	8	–	ND				22	5	13	None	47	9	26	None

*ND* not document

### PDPN correlates with better prognosis of lung Cancer

**Fig. 3:**
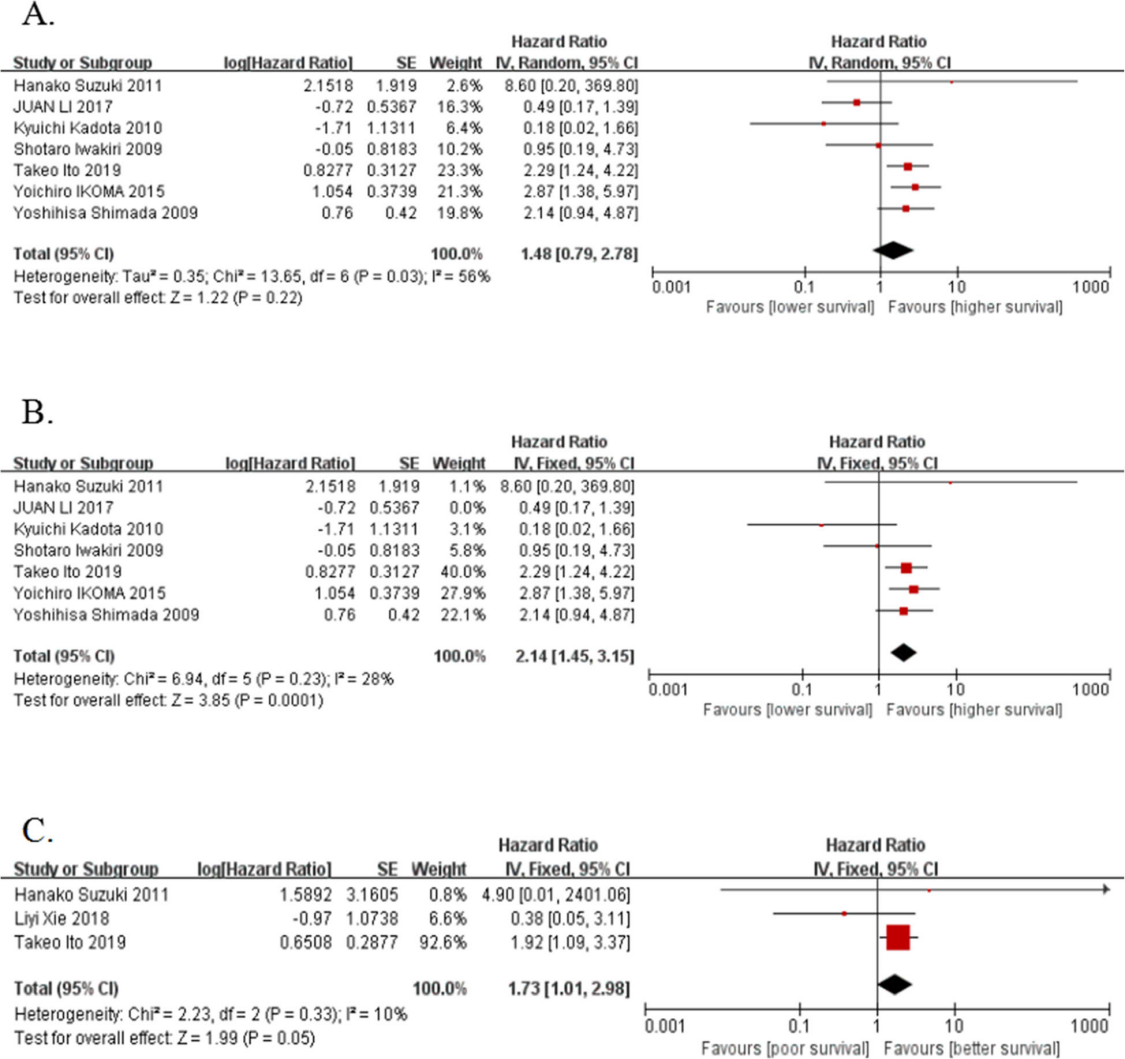
Analysis of podoplanin expression and survival of LUSC patients. Forest plot of HR for OS (**a**), OS (after deletion of Juan Li study) (**b**) and PFS (**c**) among included studies

After full-text review, 7 eligible studies including 519 LUSC patients were selected out for meta-analysis of PDPN expression with OS of lung cancer patients. Data of HR, 95%CI were extracted with the use of the methods described above. Results showed that PDPN expression has no significant associations with OS (pooled HR = 1.48, 95% CI 0.79–2.78, *P* = 0.22, random effect) (Fig. 3a). Because of the I^2^ = 56% (*P* = 0.03), which indicates that there exists heterogeneity in our results, so the sensitivity analysis was then conducted by deleting each study at on time to evaluate the stability of current result. All results were showed in Table 3. Notably, the corresponding heterogeneity has no significantly changes when deleting each single study except for the study of Juan Li (I^2^ = 28%, *P* = 0.23), which suggests that the heterogeneity of our results mostly come from the study of Juan Li. After the deletion of Juan Li study, PDPN expression showed significant associations with better OS in LUSC patients (HR = 2.14, 95% CI 1.45–3.15, *P* = 0.0001, fixed effect) (Fig. 3b).

**Table 3:** Sensitivity analysis of all 7 studies

Deleted study	No. of patients after deletion	Odds ratio		Model	Heterogeneity	
		OR (95% CI)	*P* value		I^2^	P
Hanako Suzuki 2011 [24]	479	1.40 (0.73, 2.69)	0.02	Random	61%	0.02
Juan Li 2017 [25]	437	2.14 [1.45, 3.15]	0.0001	Fixed	28%	0.23
Kyuichi Kadota 2010 [20]	469	1.74 [0.99, 3.06]	0.05	Random	47%	0.09
Shotaro Iwakiri 2009	437	1.54 [0.77, 3.08]	0.22	Random	62%	0.02
Takeo Ito 2019 [26]	383	1.25 [0.54, 2.87]	0.61	Random	61%	0.03
Yoichiro IKOMA 2015 [27]	416	1.21 [0.57, 2.60]	0.62	Random	57%	0.04
Yoshihisa Shimada 2009 [28]	357	1.30 [0.58, 2.89]	0.52	Random	63%	0.02

The meta-analysis of 3 studies showed that PDPN expression is associated with better PFS (HR = 1.73, 95% CI: 1.01–2.98, *P* = 0.05, fixed effect) (Fig. 3c), and there exist no significant heterogeneity (I^2^ = 10%).

### Publication Bias

**Fig. 4:**
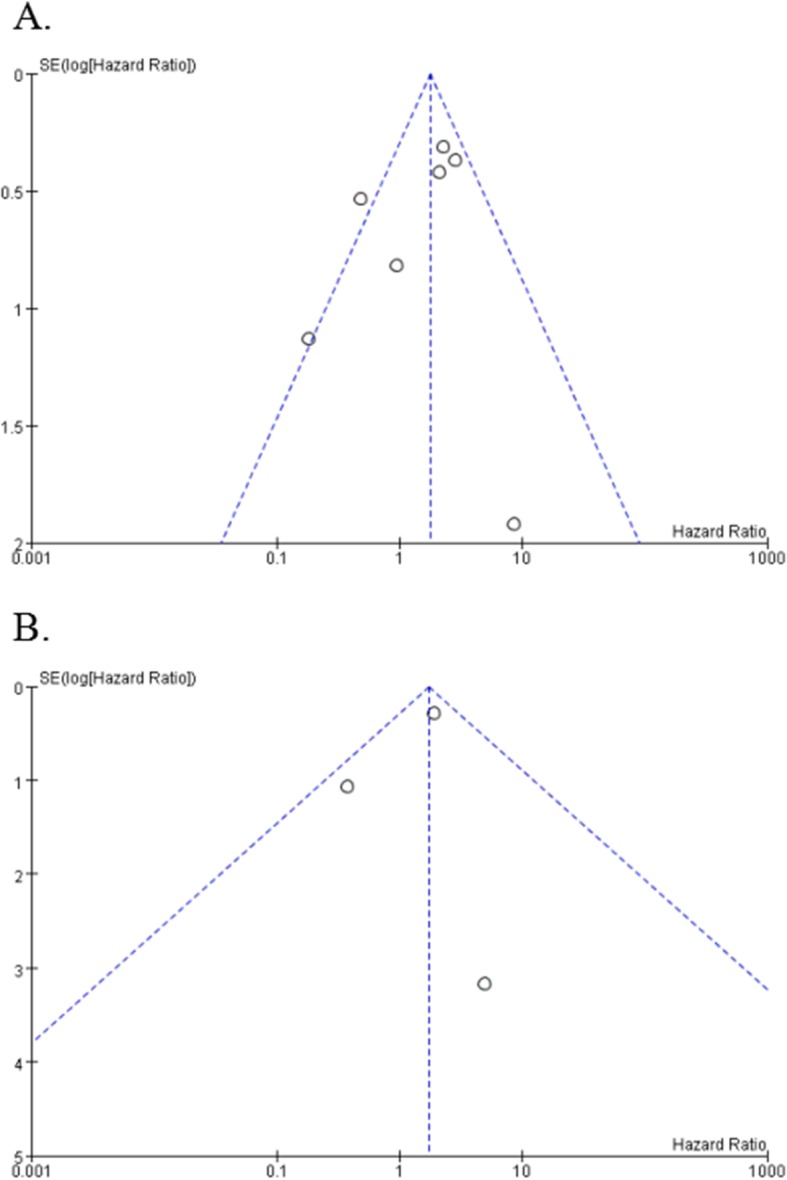
Begg’s funnel plot estimated the publication bias of the included literature for OS (**a**) and DFS (**b**)

The funnel plots illustrated no evidence of publication bias in our results (Fig. 4). No evidence for significant publication bias was found in OS (after deleting the study of Juan Li) and DFS studies.

## Discussion

The CSC markers provide an efficient therapeutic approaches for monitoring the patients’ prognosis and predicting the treatment response of cancer patients. While apart from CD133, ALDH and CD44, the validated CSC markers for lung squamous cancer is still limited [29]. As we all know, CSCs are usually located at the invasive front of tumor nest. The molecular expression pattern of cancer cells in the invading front of solid tumours is quite distinguishing from that of cells in the tumor interior [30]. Bryne M have addressed that the invasive tumour front may underlie the biological aggressiveness of carcinomas and could be taken as an vital area for tumor prognosis [31]. PDPN has also been reported to express frequently at the peripheral site of tumor nest, especially among squamous tumors including lung squamous cancer [32]. PDPN has been assumed as one of the candidate markers of cancer stem cells, associated with cancer cell invasion or migration, as well as the prognosis of specific squamous cancers [28, 33].

However, whether PDPN could be the marker of CSC in LUSC is still a question deserving further research. In our meta-analysis, among the included LUSC studies (Hanako Suzuki [24], Juan Li [25], Takeo Ito [26], Yoichiro IKOMA [27] and Yoshihisa Shimada [21]‘s study), two expression patterns for PDPN positive cases were reported and compared. One is peripheral type (P-type), and the other is diffusion type (non P-type). P-type turned to be the predominant type in PDPN positive LUSC samples (62% of the PDPN^+^ samples in Juan Li study, and 88.8% in Yoshihisa Shimada study). It all suggested that PDPN frequently located in the basal or peripheral zone of LUSC tumor nests. While from the result of survival outcomes, P-type were the independent predictor of patients with better OS (IKOMA, HR, 2.443; 95% CI, 1.202–4.964, *P* = 0.014; Shimada, 5-year overall survival rates 71.7% (P type) versus 54.8% (non-P type), *P* = 0.043) [27]. It all suggested that SqCC with the P-type pattern may indicate lower biological aggressiveness.

In regard to this interesting results, we think there are several ways to understand it. Firstly, as we all know, if the morphology and function of a tumor are close to normal tissue, it indicates high degree of differentiation or a good differentiation [34]. Shimada speculated that the P-type pattern maybe a well-organized tumor group, just like the structure of epithelial tissue, whereas SqCC with an non-P type is a disordered tumor group in terms of the developmental hierarchy. It suggests that P-type may indicates a higher differentiation and a more organized tumor group. As P-type is the predominant type of PDPN positive LUSC, we could conclude that PDPN positive LUSC may indicate higher differentiation. Actually from our results, PDPN do have significant correlations with tumor better differentiation in LUSC (HR = 2.14, 95% CI = 1.34–3.43, *P* = 0.002). Oksana Kowalczuk’s study also manifested that PDPN transcriptional downregulation was more significant in high-graded tumors (G3 or G4) compared with low-graded ones (G1 or G2) (*P* = 0.049) among I-III lung cancer patients [35], which coincides with our results.

Moreover, our results showed that expression of PDPN do not associated with EMT process including TNM stage, vascular invasion, lymphatic invasion, lymph node metastasis and pleural metastasis of tumor. In Takashi Saku’s study, they demonstrated that PDPN contribution to cell proliferation has proved only to be a secondary event to cell adhesion, and the present PDPN inhibition by siRNA did not affect cell migration [36]. PDPN has been known as the specific marker for lymphatic vessels, for its role in lymphangiogenesis [37]. Ezrin and moesin, which belong to the ERM (ezrin, radixin, moesin) protein family, could bind with the cytosolic domain of PDPN, and then rearrange the actin cytoskeleton, which may involves in lymphangiogenesis, lymph node metastasis and epithelial-mesenchymal transition (EMT) [38]. However, in both vivo and vitro in lung cancer, Hanako Suzuki revealed that exogenous PDPN had no influence on tumor growth, and PDPN significantly restrained axillary lymph node metastasis associated with the suppression of lymphangiogenesis through the downregulation of EBC-1-derived VEGF-C mRNAs [33]. According to those results, the value of PDPN expression as a marker for cancer stem cells in LUSC should be critically evaluated in future studies.

Sensitivity analysis showed that the heterogeneity of our meta-analysis mainly came from Juan Li’s study (I^2^ = 56%, *P* = 0.03). We think there exists several possible reasons. First, in the study of Juan Li, they included IV patients, while other studies only contains I-III LUSC patients. Even though, we couldn’t get the exact number of IV patients involved, but different cancer stage will results in completely different survival results. Another, the cut-off value of positive and negative PDPN expression in Juan Li’s study is different from other studies. In Juan Li’s study, only >80% membrane immunohistochemical staining were conceived as PDPN positive, while in other studies the cut-off value is around 10–20%. Currently, there is no standard criteria for positive immunohistochemical staining of PDPN. There is an urgent need for unified division standard for ‘positive’ and ‘negative’ PDPN according to its clinical role in survival benefits as the further research develops. Last, in our study low expression of PDPN correlated with low differentiation of LUSC, which means more malignancy and more resistance to chemo-radio treatments. Thus, it could explain why low PDPN may predict poor survival in LUSC.

There are also limitations in this meta-analysis. First, the number of included studies, as well as the included LUSC patients in each study, is relatively small. Thus, those factors may reduce the power and accuracy of this meta-analysis. Second, the survival outcomes (OS and PFS) were based on unadjusted HRs. Third, as discussed above, the thresholds of the cut-off value is not all the same. Thus, the uniform definition of positive PDPN expression is more helpful to obtain more accurate results.

## Conclusion

This study supports that PDPN could be a useful of better prognostic maker and indicates better differentiation for LUSC patients, and the value of PDPN expression as a marker for cancer stem cells in LUSC should be critically evaluated in future studies. Further researches should be focused on unified cut-off standard to detect the expression of PDPN, and its unique expression type (P-type or non P-type) in tumor, thus to undermine the mechanism of PDPN in squamous lung cancer progression.

## Data Availability

All data generated or used during the study appear in the submitted article.

## Abbreviations

NSCLC: Non-small cell lung cancer
LUSC: Lung squamous cell carcinoma
CSCs: Cancer stem cells
PDPN: Podoplanin
SCC: Squamous cell cancers
cSCC: Cutaneous squamous cell carcinoma
PFS: Progression-free survival
OS: Overall survival
